# Supplementary material for: Generation of Transgenic Sperm Expressing GFP by Lentivirus Transduction of Spermatogonial Stem Cells In Vivo in Cynomolgus Monkeys
Source: Vet Sci. 2023 Feb 1;10(2):104. doi: 10.3390/vetsci10020104 (PMC9966439; doi:10.3390/vetsci10020104)
Supplement: Supplementary file 1 [file vetsci-10-00104-s001.zip › Figures S1¿CS8.pdf]

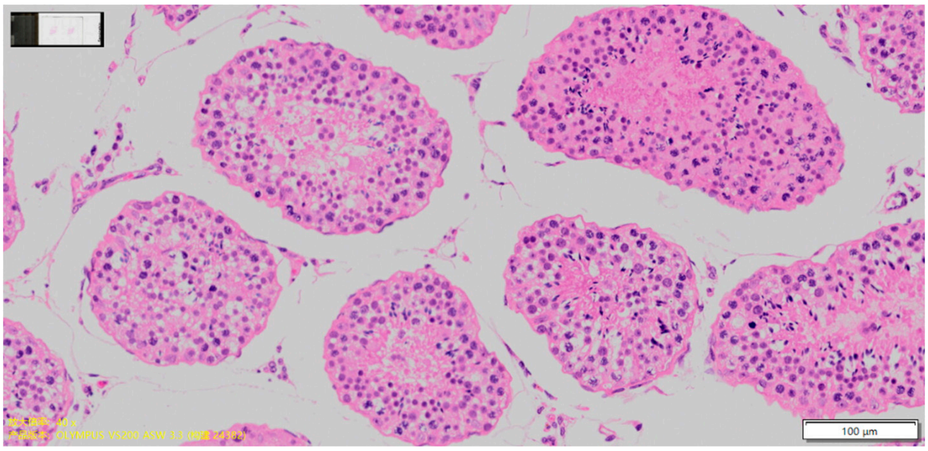

Figure S1. Testis pre-EGFP: Testicular tissue morphology at different microscope magnifications before EGF lentivirus injection. (n=3).

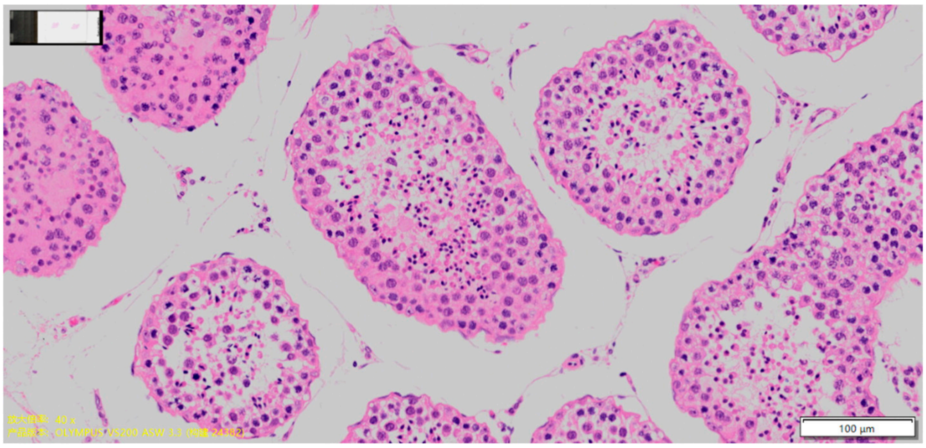

Figure S2. Testis after-EGFP: Testicular tissue morphology at different microscope magnifications after EGFP lentivirus injection.

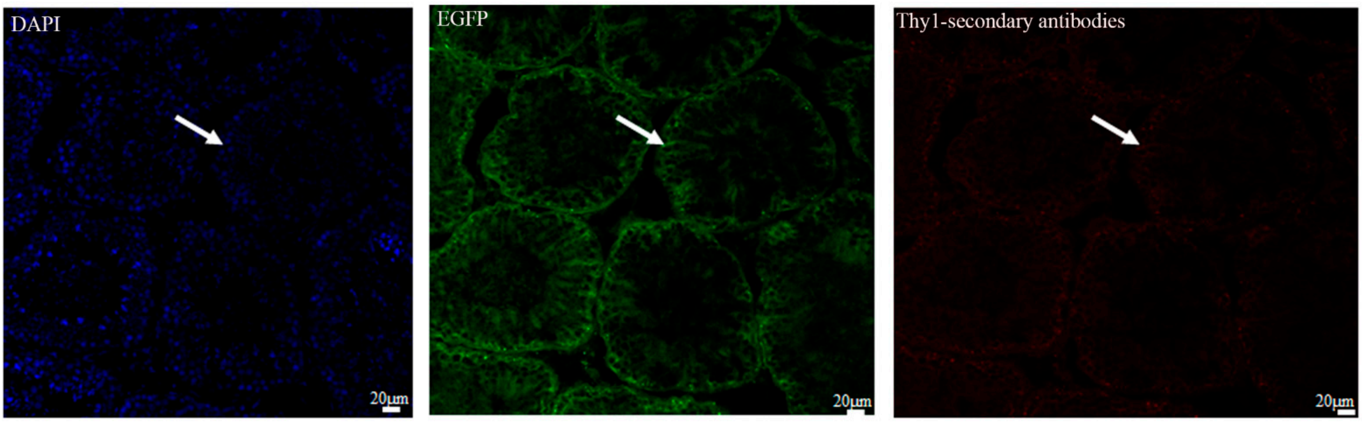

Figure S3. Immunofluorescence examination: We found specific immunolabeling was observed around the wall of the seminiferous tubules. In order to confirm that Thy1 could be the marker of SSCs and not the result of secondary antibodies, Thy1-econdary antibodies were stained for the EGFP-transfected seminiferous tubules and no fluorescence was found. Therefore, Thy1 could be used as the marker of SSCs.

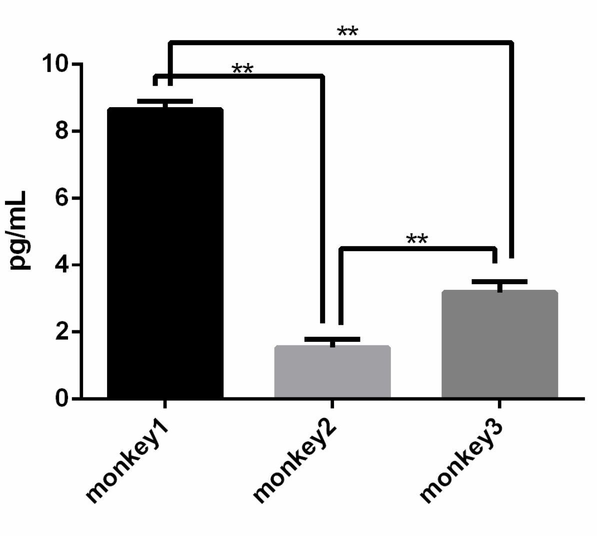

Figure S4. q-PCR: The amount of EGFP integration in the genome, the bar chart represents the amount of EGFP expression in the 3 monkey sperms transducted with EGFP lentivirus relative to the control group. All data expressed as the means ± SEM. Asterisks stands for significance: * < 00.5, ** < 0.01, *** < 0.001, **** < 0.0001, (n = 3).

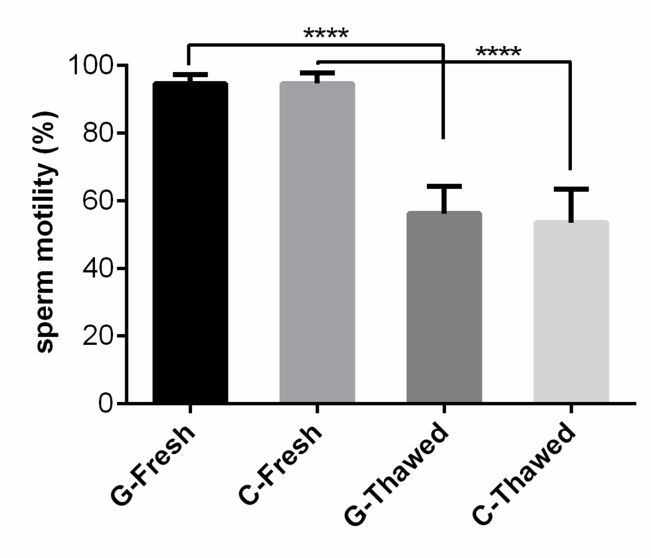

Figure S5. Evaluation of sperm motility with transgenic sperm carrying EGFP gene. G-Fresh: fresh sperm with transgenic sperm carrying EGFP gene, C-Fresh: Control group of fresh sperm (fresh sperm with no transgenic), all data expressed as the means ± SEM; (P < 0.05). Asterisks stands for significance: * < 00.5, ** < 0.01, *** < 0.001, **** < 0.0001, (n = 3).

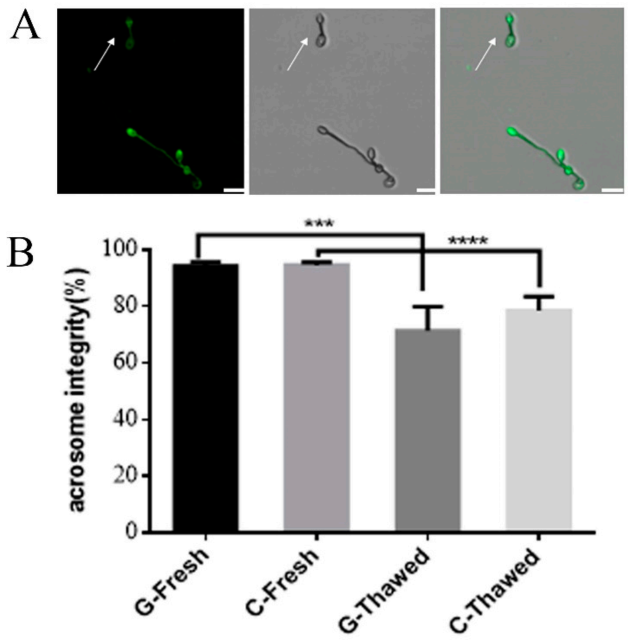

Figure S6. Evaluation of acrosome integrity. (**A**) Frozen−thawed sperm cells with intact and reacted acrosomes; Arrow indicates an acrosome damaged sperm, sperm with little or no green fluorescent staining in the front of the head were considered to have acrosomal sperm impaired. (**B**) Acrosome integrity was analyzed statistically by histogram. At least 200 sperm per semen sample were evaluated for this staining. G-Fresh: fresh sperm with transgenic sperm carrying EGFP gene, C-Fresh: Control group of fresh sperm (fresh sperm with no transgenic), all data expressed as the means ± SEM; (P < 0.05). * stands for significance. * stands for significance: Asterisks stands for significance: * < 00.5, ** < 0.01, *** < 0.001, **** < 0.0001, (*n* = 3).

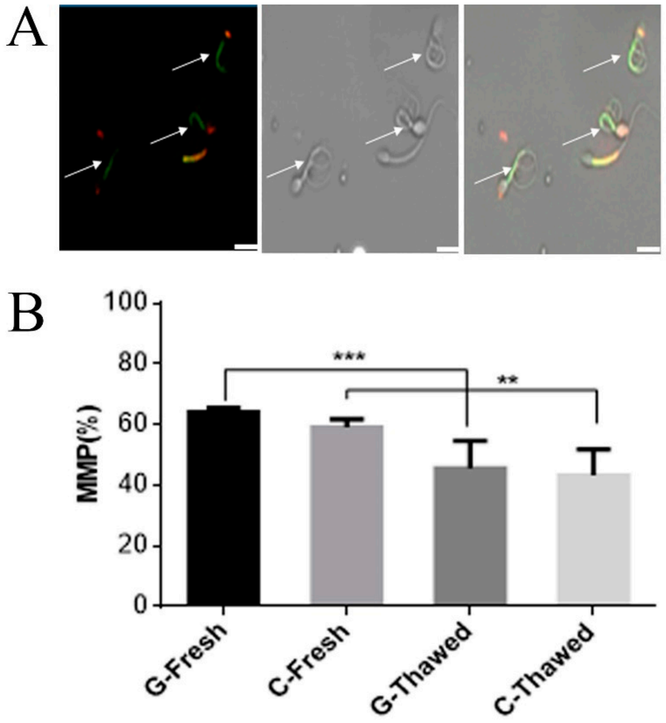

Figure S7. Evaluation of mitochondrial membrane potential. (**A**) Frozen−thawed sperm cells with mitochondrial membrane potential; Sperm with intact mitochondria fluoresce in orange and yellow. In contrast, sperm with damaged mitochondria emit green fluorescence. Damaged sperm mitochondrial membrane potential is shown by arrows. At least 200 sperm in each sample were

evaluated for mitochondrial potential using a fluorescent staining procedure. (**B**) Mitochondrial membrane potential was analyzed statistically by histogram. G-Fresh: fresh sperm with transgenic sperm carrying EGFP gene, C-Fresh: Control group of fresh sperm (fresh sperm with no transgenic), all data expressed as the means ± SEM; (P < 0.05). Asterisks stands for significance: * < 00.5, ** < 0.01, *** < 0.001, **** < 0.0001, (*n* = 3).

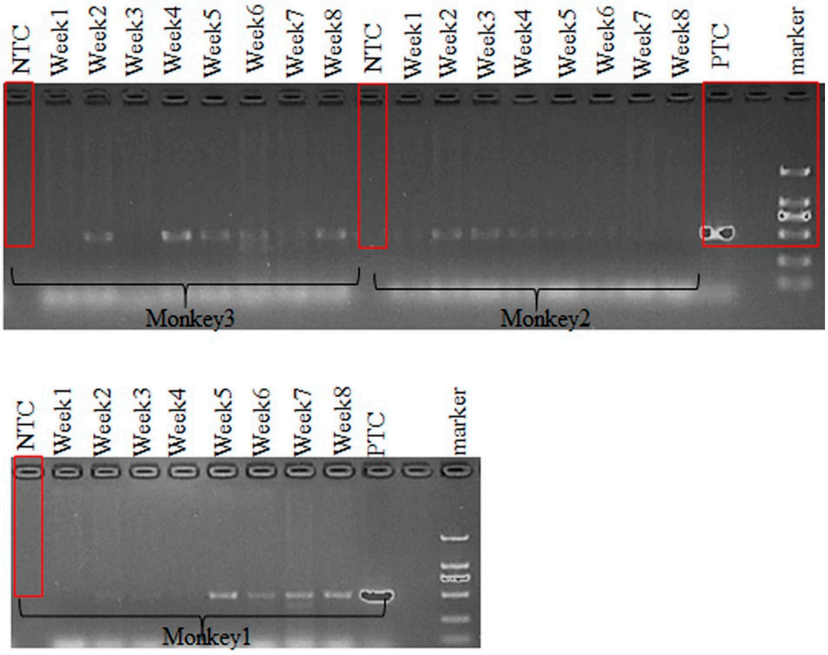

Figure S8. NTC: Water replaced the sperm sample as a negative control; PTC: EGFP lentivirus replaced the sperm sample as a positive control. The red box represents the cut in manuscript.
